# Differences in Stage of Cancer at Diagnosis, Treatment, and Survival by Race and Ethnicity Among Leading Cancer Types

**DOI:** 10.1001/jamanetworkopen.2020.2950

**Published:** 2020-04-08

**Authors:** Chenyue Zhang, Chenxing Zhang, Qingliang Wang, Zhenxiang Li, Jiamao Lin, Haiyong Wang

**Affiliations:** 1 Department of Integrated Therapy, Fudan University Shanghai Cancer Center, Shanghai Medical College, Shanghai, China; 2 Department of Nephrology, Shanghai Children’s Medical Center, Shanghai Jiao Tong University School of Medicine, Shanghai, China; 3 Department of Medical Affairs, Qilu Hospital of Shandong University, Jinan, China; 4 Department of Internal Medicine–Oncology, Shandong Cancer Hospital and Institute, Shandong First Medical University and Shandong Academy of Medical Sciences, Jinan, Shandong, China

## Abstract

**Importance:**

Information about stage of cancer at diagnosis, use of therapy, and survival among patients from different racial/ethnic groups with 1 of the most common cancers is lacking.

**Objective:**

To assess stage of cancer at diagnosis, use of therapy, overall survival (OS), and cancer-specific survival (CSS) in patients with cancer from different racial/ethnic groups.

**Design, Setting, and Participants:**

This cohort study included 950 377 Asian, black, white, and Hispanic patients who were diagnosed with prostate, ovarian, breast, stomach, pancreatic, lung, liver, esophageal, or colorectal cancers from January 2004 to December 2010. Data were collected using the Surveillance, Epidemiology, and End Results (SEER) database, and patients were observed for more than 5 years. Data analysis was conducted in July 2018.

**Main Outcomes and Measures:**

Multivariable logistic and Cox regression were used to evaluate the differences in stage of cancer at diagnosis, treatment, and survival among patients from different racial/ethnic groups.

**Results:**

A total of 950 377 patients (499 070 [52.5%] men) were included in the study, with 681 251 white patients (71.7%; mean [SD] age, 65 [12] years), 116 015 black patients (12.2%; mean [SD] age, 62 [12] years), 65 718 Asian patients (6.9%; mean [SD] age, 63 [13] years), and 87 393 Hispanic patients (9.2%; mean [SD] age, 61 [13] years). Compared with Asian patients, black patients were more likely to have metastatic disease at diagnosis (odds ratio [OR], 1.144; 95% CI, 1.109-1.180; *P* < .001). Black and Hispanic patients were less likely to receive definitive treatment than Asian patients (black: adjusted OR, 0.630; 95% CI, 0.609-0.653; *P* < .001; Hispanic: adjusted OR, 0.751; 95% CI, 0.724-0.780; *P* < .001). White, black, and Hispanic patients were more likely to have poorer CSS and OS than Asian patients (CSS, white: adjusted HR, 1.310; 95% CI, 1.283-1.338; *P* < .001; black: adjusted HR, 1.645; 95% CI, 1.605-1.685; *P* < .001; Hispanic: adjusted HR, 1.300; 95% CI, 1.266-1.334; *P* < .001; OS, white: adjusted HR, 1.333; 95% CI, 1.310-1.357; *P* < .001; black: adjusted HR, 1.754; 95% CI, 1.719-1.789; *P* < .001; Hispanic: adjusted HR, 1.279; 95% CI, 1.269-1.326; *P* < .001).

**Conclusions and Relevance:**

In this study of patients with 1 of 9 leading cancers, stage at diagnosis, treatment, and survival were different by race and ethnicity. These findings may help to optimize treatment and improve outcomes.

## Key Points

**Question:**

Do stage of cancer at diagnosis, use of definitive therapy, and survival differ by race/ethnicity among patients with 1 of the most common cancers?

**Findings:**

In this cohort study of 950 377 patients with cancer, stage at diagnosis, treatment, and survival varied by race and ethnicity. Overall, compared with Asian patients, black patients were more likely to have metastatic disease at diagnosis, black and Hispanic patients were less likely to receive definitive treatment, and white, black, and Hispanic patients had worse odds of cancer-specific and overall survival.

**Meaning:**

The findings of this study may lead to different management strategies based on race and ethnicity to improve outcomes.

## Teaser

This cohort study assesses the stage of cancer at diagnosis, use of therapy, overall survival, and cancer-specific survival among patients with cancer from different racial/ethnic groups.

## Introduction

Cancer is the leading cause of morbidity and mortality worldwide, and it differs greatly among racial and ethnic groups.^1,2,3^ The discrepancy is multifactorial and could be attributed to tobacco or alcohol consumption, obesity, genetic susceptibility to cancer, and access to high-quality health care.^4,5^

Most studies have focused on the role of race and ethnicity on survival in 1 type of malignant neoplasm, failing to explore their associations with outcomes for an overall set of cancers.^6,7^ In addition, the role of race/ethnicity differs in these studies, which have not reached a consistent conclusion.^8,9,10^ Particularly, there is a lack of research analyzing stage at diagnosis, use of therapy, and prognosis among the leading cancers by race/ethnicity. Therefore, we included the 9 most common cancers in 1 analysis to test these differences, which could be helpful for optimizing treatments among patients from different racial/ethnic groups.

We aimed to develop a comprehensive summary of cancer metastasis, treatment, and survival in the United States among patients patients from different racial/ethnic groups, which could serve as a reference source. Related health strategies to promote primary prevention, cancer screening, early diagnosis, and treatment options should be specifically targeted to improve cancer survival among patients patients from different racial/ethnic groups in the United States.

## Methods

### Patient Selection

With approval from the review board of Shandong Cancer Hospital and Institute, we explored the outcomes of 9 leading cancers in the United States using data from the Surveillance, Epidemiology, and End Results (SEER) database. An exemption of informed consent was granted by the ethics committee of Shandong Cancer Hospital and Institute because the SEER database is open access. This study followed the Strengthening the Reporting of Observational Studies in Epidemiology (STROBE) reporting guideline.

The SEER database covers cancer incidence data from population-based cancer registries from 18 geographically diverse populations that represent rural, urban, and regional populations, accounting for 34.6% of the US population. Patients diagnosed from January 2004 to December 2010 with a leading cancer (ie, prostate, ovarian, breast, stomach, pancreatic, lung, liver, esophageal, or colorectal cancer) were collected. Patients were observed for more than 5 years. The SEER database includes information on age, sex, clinical stage, treatment, and tumor category; SEER is a public access database, and patients’ corresponding details were retrieved with the use of SEER*Stat version 8.3.5 software (National Cancer Institute).

Upon initial treatment, patients were followed up with for detailed information.^11^ We determined that 2004 was the first year that many covariates were introduced.^12^ Patients with the following features were excluded: (1) younger than 18 years at diagnosis, (2) those whose diagnosis was made at autopsy, and (3) those with an earlier diagnosis of another malignant neoplasm, incomplete clinical information, or unknown causes of death. A total of 950 377 patients were included in the final cohort.

### Race/Ethnicity Classification and Variables

Race and ethnicity were self-reported. Patients were divided into the 4 following categories: non-Hispanic white (white), non-Hispanic black (black), non-Hispanic Asian or Pacific Islander (Asian), and Hispanic. For each patient, age at diagnosis, sex, tumor stage, node stage, metastasis stage upon diagnosis, treatment, and tumor category were assigned.

### Vital Status

The SEER public access database and patients’ corresponding details were retrieved with the use of SEER*Stat software version 8.3.5 (National Cancer Institute), which covered data from 2004 to 2015. We only included patients diagnosed from 2004 to 2010 to guarantee that all included patients could be observed for more than 5 years. Data analysis was performed in July 2018.

### Statistical Analysis

Descriptive statistics *t *test or χ^2^ test were used to compare patients’ baseline characteristics, as follows: age at diagnosis, sex, TNM stage, treatment received, and tumor category. For each racial/ethnic group, differences in demographic and tumor characteristics were examined by χ^2^ tests for categorical variables and *t* tests for continuous variables. Multivariable logistic regression was used to measure the association of race/ethnicity with stage at diagnosis after adjustment for demographic factors. Stage at diagnosis was categorized as metastatic disease and nonmetastatic disease. Tumor and nodal stage were refereed and determined by the American Joint Committee on Cancer Staging Manual.^12^

Among the 950 377 patients included, 783 113 patients received therapy, which was divided as follows: (1) patients with prostate, lung, pancreatic, liver/intrahepatic bile duct (IHBD), or esophageal cancer undergoing surgery and/or radiation therapies and 2) patients with breast, stomach, colorectal, ovarian, or gastric cancer undergoing surgery.

Multivariable logistic regression was used to assess odds ratios (ORs) among patients patients from different racial/ethnic groups who potentially had metastatic disease and/or received treatment. We also computed 95% CIs for ORs. Cox proportional hazards multivariable regression was used to evaluate the association of race and ethnicity with overall survival (OS) and cancer-specific survival (CSS) by calculating hazard ratios (HRs) with other factors adjusted. In addition, 95% CIs for HRs were generated.

Statistical significance was set at *P* < .05, and all tests were 2-tailed. Statistical analyses were conducted with SAS version 9.3 (SAS Institute).

## Results

### Patient Characteristics

We identified 950 377 patients diagnosed between 2004 and 2010 in the SEER database with known races and ethnicities. The demographic and clinical characteristics of the study population according to racial/ethnic group are presented in Table 1. The mean (SD) age at diagnosis was lowest among Hispanic patients (87 393 patients [9.2%]; 61 [13] years) and highest among white patients (681 251 patients [71.7%]; 65 [12] years). The mean (SD) age of black patients (116 015 [12.2%]) and Asian patients (65 718 [6.9%]) was 62 (12) years and 63 (13) years, respectively. There was a total of 499 070 (52.5%) men and 451 307 (47.5%) women. Except among Asian patients (for whom women outnumbered men, with 34 828 women [53.0%] and 30 890 men [47.0%]), white, black, and Hispanic patients had a higher percentage of male patients. We analyzed tumor stages among the 4 racial/ethnic groups at diagnosis. Overall, a significant difference of T stage was found among patients from the patients from the different racial/ethnic groups. A total of 248 669 white patients (36.5%) had stage T1 disease at diagnosis. The percentages of stage T1 disease among black, Asian, and Hispanic patients were 35.2% (40 821), 36.7% (24 078), and 35.0% (30 597), respectively (*P* < .05). The percentages of white, black, Asian, and Hispanic patients with stage T2 disease were 33.7% (229 729), 34.0% (39 408), 30.5% (20 057), and 34.5% (30 146), respectively (*P* < .05). Table 1 also presents the ratios of T3 and T4 stages by race/ethnicity. Significant differences were found among white, black, Asian, and Hispanic patients for N and M stage as well (eg, N0: white, 473 934 [69.6%]; black, 80 412 [69.3%]; Asian, 43 804 [66.7%]; Hispanic, 60 143 [68.8%]; *P* < .05). Tumor distribution was demonstrated across the 4 racial/ethnic groups; the 2 cancers with the highest incidence rates among the 9 cancers on the list were prostate cancer (white, 203 295 [29.8%]; black, 42 314 [36.5%]; Asian, 13 497 [20.5%]; Hispanic, 25 635 [29.3%]) and breast cancer (white, 181 887 [26.7%]; black, 26 305 [22.7%]; Asian, 19 666 [29.9%]; Hispanic, 25 680 [29.4%]). White and black patients had the highest incidences of prostate cancer, followed by breast cancer. Among Asian and Hispanic patients, breast cancer accounted for the most tumors, followed by prostate cancer.

**Table 1.** Baseline Demographic and Clinical Characteristics

Characteristic	No. (%)^a^			
	White patients (n = 681 251)	Black patients (n = 116 015)	Asian patients (n = 65 718)	Hispanic patients (n = 87 393)
Age, mean (SD), y	65 (12)	62 (12)	63 (13)	61 (13)
Sex				
Men	356 556 (52.3)	66 888 (57.7)	30 890 (47.0)	44 736 (51.2)
Women	324 695 (47.7)	49 127 (42.3)	34 828 (53.0)	42 657 (48.8)
T stage				
1	248 669 (36.5)	40 821 (35.2)	24 078 (36.7)	30 597 (35.0)
2	229 729 (33.7)	39 408 (34.0)	20 057 (30.5)	30 146 (34.5)
3	116 283 (17.1)	19 570 (16.9)	12 553 (19.1)	16 662 (19.1)
4	86 570 (12.7)	16 216 (14.0)	9030 (13.7)	9988 (11.4)
N stage				
0	473 934 (69.6)	80 412 (69.3)	43 804 (66.7)	60 143 (68.8)
1	103 203 (15.1)	17 714 (15.3)	11 415 (17.3)	15 384 (17.6)
2	81 607 (12.0)	13 736 (11.8)	7892 (12.0)	9177 (10.5)
3	22 507 (3.3)	4153 (3.6)	2607 (4.0)	2689 (3.1)
M stage				
0	577 008 (84.7)	96410 (83.1)	55 126 (83.9)	75 134 (86.0)
1	104 243 (15.3)	19605 (16.9)	10 592 (16.1)	12 259 (14.0)
Treatment				
Yes	566 224 (83.1)	91 009 (78.4)	54 466 (82.9)	71 414 (81.7)
No	115 027 (16.9)	25 006 (21.6)	11 252 (17.1)	15 979 (18.3)
Tumor category				
Prostate	203 295 (29.8)	42 314 (36.5)	13 497 (20.5)	25 635 (29.3)
Ovarian	15 982 (2.3)	1645 (1.4)	1763 (2.7)	2536 (2.9)
Breast	181 887 (26.7)	26 305 (22.7)	19 666 (29.9)	25 680 (29.4)
Stomach	9957 (1.5)	2557 (2.2)	3170 (4.8)	3465 (4.0)
Pancreatic	18 106 (2.7)	3174 (2.7)	1865 (2.8)	2606 (3.0)
Lung	126 713 (18.6)	19 287 (16.6)	10 195 (15.5)	9052 (10.4)
Liver/IHBD	7987 (1.2)	1996 (1.7)	2857 (4.3)	2616 (3.0)
Esophageal	9387 (1.4)	1345 (1.2)	563 (0.9)	846 (1.0)
Colorectal	107 937 (15.8)	17 392 (15.0)	12 142 (18.5)	14 957 (17.1)

Abbreviation: IHBD, intrahepatic bile duct.

^a^ All comparisons of characteristics of white, black, Asian, and Hispanic patients had *P* < .05.

### Difference in Stage at Diagnosis by Race and Ethnicity Among the Leading Cancers

**Figure 1.**
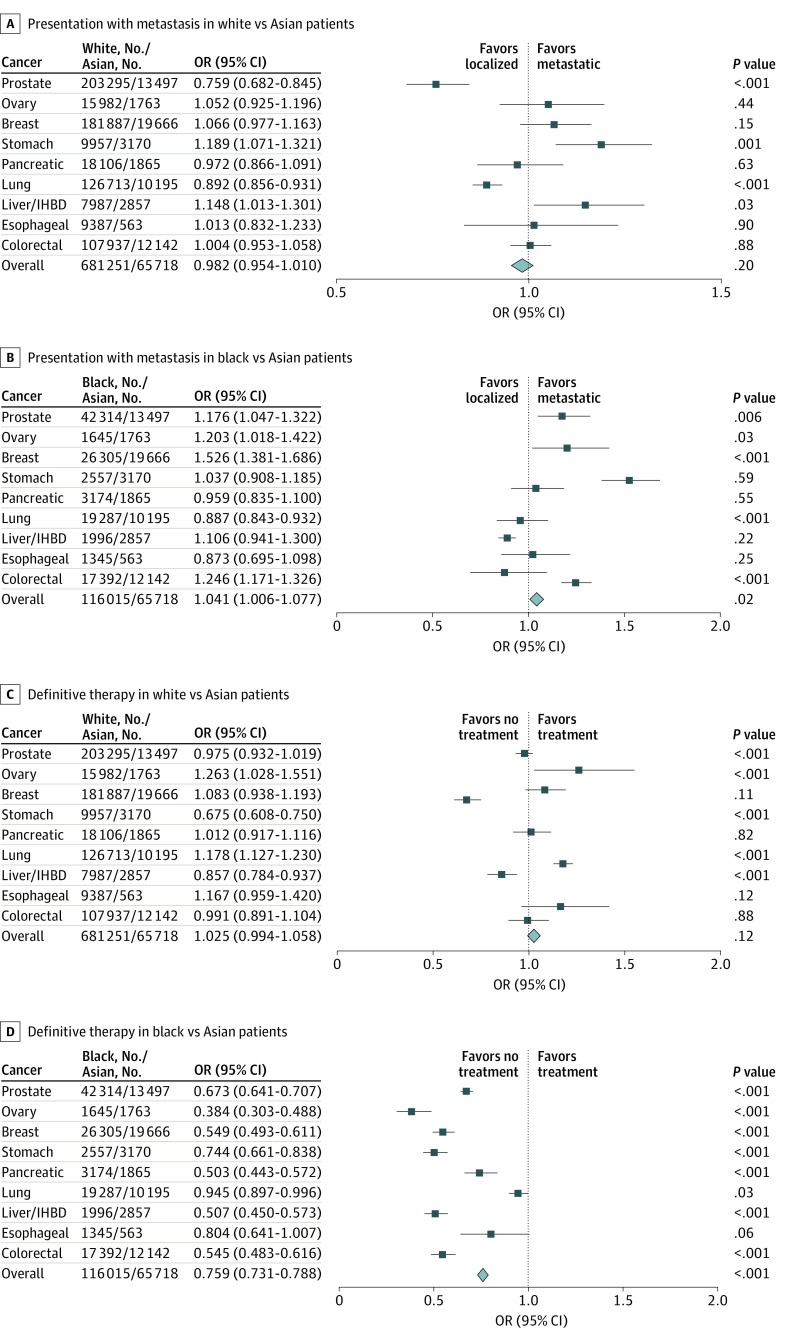
Difference in Stage at Diagnosis and Treatment Between White, Black, and Asian Patients With Leading Cancers Sex-specific cancers, such as prostate, breast, and ovarian cancers, were not included in the overall analysis. IHBD indicates intrahepatic bile duct; and OR, odds ratio.

To compare stage at diagnosis in different racial and ethnic groups, the cohort was divided into 4 subgroups (white, black, Asian, and Hispanic). Figure 1 and eFigure 1 in the Supplement show the ORs of metastasis for all 9 cancers among white, black, and Hispanic patients compared with Asian patients. After adjusting for demographic characteristics, white patients were more likely than Asian patients to have metastatic stomach cancer (OR, 1.189; 95% CI, 1.071-1.321; *P* = .001) and liver and/or IHBD cancer (OR, 1.148; 95% CI, 1.013-1.301; *P* = .03) (Figure 1A). Compared with Asian patients, black patients were more likely to have metastatic prostate cancer (OR, 1.176; 95% CI, 1.047-1.322; *P* = .006), ovarian cancer (OR, 1.203; 95% CI, 1.018-1.422; *P* = .03), breast cancer (OR, 1.526; 95% CI, 1.381-1.686; *P* < .001), and colorectal cancer (OR, 1.246; 95% CI, 1.171-1.326; *P* < .001) (Figure 1B). Compared with Asian patients, Hispanic patients showed metastatic tendency only in stomach cancer (OR, 1.206; 95% CI, 1.066-1.364; *P* = .003) among the 9 leading cancers (eFigure 1 in the Supplement). As shown in Table 2, after adjusting for demographic characteristics, black patients were more likely to develop metastatic disease than Asian patients (OR, 1.144; 95% CI, 1.109-1.180; *P* < .001).

**Table 2.** Associations of Race/Ethnicity With Presentation With Metastatic Disease and Use of Definitive Therapy

Population	Metastasis at diagnosis		Definitive treatment	
	OR (95% CI)	*P* value	OR (95% CI)	*P* value
Asian	1 [Reference]	NA	1 [Reference]	NA
White	0.986 (0.960-1.011)	.27	1.027 (0.996-1.059)	.09
Black	1.144 (1.109-1.180)	<.001	0.630 (0.609-0.653)	<.001
Hispanic	1.002 (0.970-1.036)	.89	0.751 (0.724-0.780)	<.001

Abbreviations: NA, not applicable; OR, odds ratio.

### Difference in Treatment by Race and Ethnicity Among the Leading Cancers

Figure 1 and eFigure 1 in the Supplement show the rates of treatment for the 9 leading cancers among white, black, and Hispanic patients compared with Asian patients. Results demonstrated that white patients were more likely to receive definitive therapy only in ovarian cancer (OR, 1.263; 95% CI, 1.028-1.551; *P* < .001) and lung cancer (OR, 1.178; 95% CI, 1.127-1.230; *P* < .001) (Figure 1C). Results revealed that black patients were less likely to receive treatment in all cancers except esophageal cancer (eg, ovarian cancer: OR, 0.384; 95% CI, 0.303-0.488; *P* < .001; esophageal cancer: OR, 0.804; 95% CI, 0.641-1.007; *P* = .06) compared with Asian patients (Figure 1D). Hispanic patients were less likely to receive treatment for all leading cancers except breast and esophageal cancer (eg, liver and/or IHBD cancer: OR, 0.543; 95% CI, 0.486-0.607; P < .001; breast cancer: OR, 0.896; 95% CI, 0.796-1.008; *P* = .07; esophageal cancer: OR, 0.899; 95% CI, 0.704-1.147; *P* = .39) compared with Asian patients (eFigure 1 in the Supplement). As shown in Table 2, black and Hispanic patients were less likely to receive definitive treatment than Asian patients (black: adjusted OR, 0.630; 95% CI, 0.609-0.653; *P* < .001; Hispanic: adjusted OR, 0.751; 95% CI, 0.724-0.780; *P* < .001).

### Difference in CSS and OS by Race and Ethnicity Among the Leading Cancers

**Figure 2.**
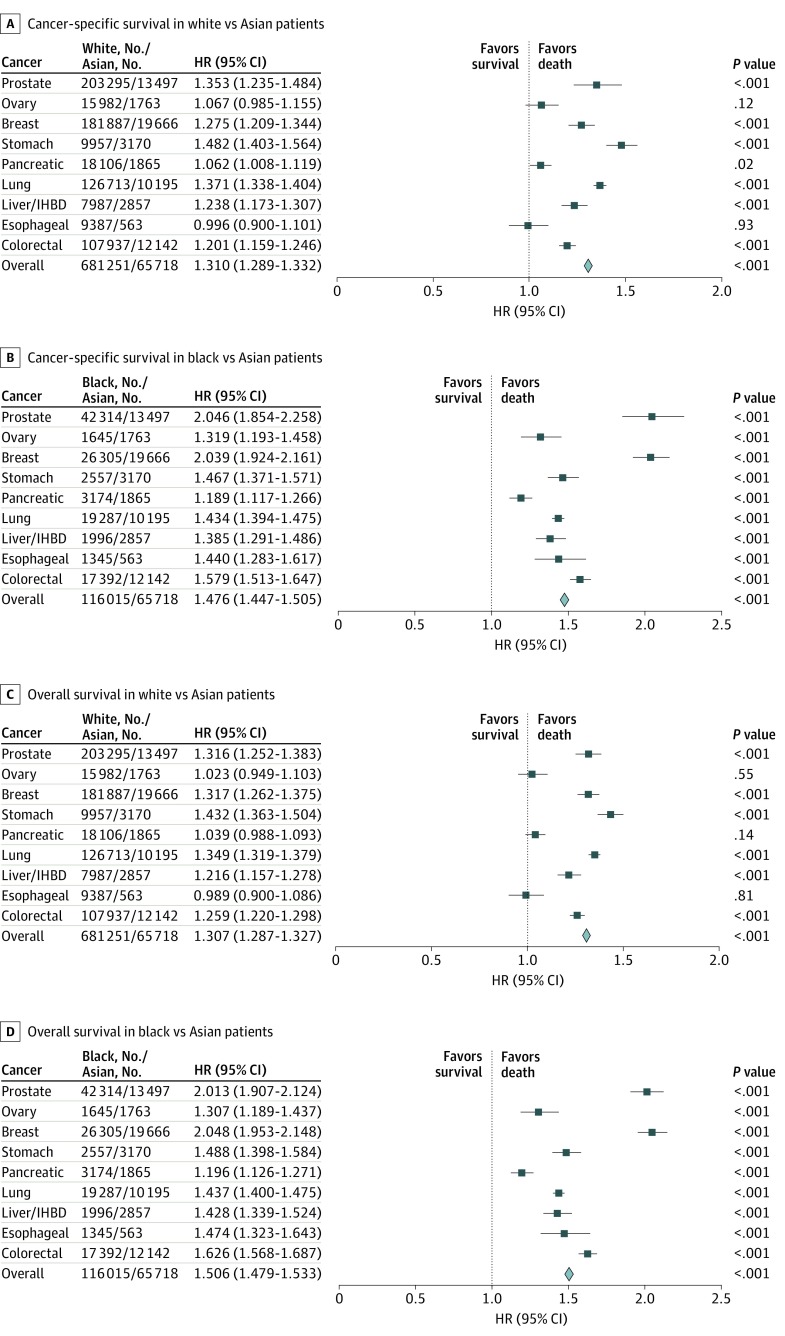
Difference in Cancer-Specific Survival and Overall Survival Between White, Black, and Asian Patients With Leading Cancers Sex-specific cancers, such as prostate, breast, and ovarian cancers, were not included in the overall analysis. HR indicates hazard ratio; and IHBD, intrahepatic bile duct.

Figure 2 and eFigure 2 in the Supplement show CSS and OS for all 9 cancers among white, black, and Hispanic patients compared with Asian patients. As shown in Figure 2A, compared with Asian patients, white patients had poorer CSS for prostate cancer (HR, 1.353; 95% CI, 1.235-1.484; *P* < .001), breast cancer (HR, 1.275; 95% CI, 1.209-1.344; *P* < .001), stomach cancer (HR, 1.482; 95% CI, 1.403-1.564; *P* < .001), pancreatic cancer (HR, 1.062; 95% CI, 1.008-1.119; *P* = .02), lung cancer (HR, 1.371; 95% CI, 1.338-1.404; *P* < .001), liver and/or IHBD cancer (HR, 1.238; 95% CI, 1.173-1.307; *P* < .001), and colorectal cancer (HR, 1.201; 95% CI, 1.159-1.246; *P* < .001). White patients also had lower OS than Asian patients for prostate cancer (HR, 1.316; 95% CI, 1.252-1.383; *P* < .001), breast cancer (HR, 1.317; 95% CI, 1.262-1.375; *P* < .001), stomach cancer (HR, 1.432; 95% CI, 1.363-1.504; *P* < .001), lung cancer (HR, 1.349; 95% CI, 1.319-1.379; *P* < .001), liver and/or IHBD cancer (HR, 1.216; 95% CI, 1.157-1.278; *P* < .001), and colorectal cancer (HR, 1.259; 95% CI, 1.220-1.298; *P* < .001) (Figure 2C). Compared with Asian patients, black patients had worse CSS and OS in all 9 cancers (eg, prostate cancer, CSS: HR, 2.046; 95% CI, 1.854-2.258; *P* < .001; OS: HR, 2.013; 95% CI, 1.907-2.124; *P* < .001) (Figure 2B and Figure 2D). Hispanic patients had poorer CSS for prostate cancer (HR, 1.357; 95% CI, 1.220-1.510; *P* < .001), breast cancer (HR, 1.339; 95% CI, 1.258-1.426; *P* < .001), stomach cancer (HR, 1.300; 95% CI, 1.218-1.387; *P* < .001), lung cancer (HR, 1.262; 95% CI, 1.221-1.305; *P* < .001), liver and/or IHBD cancer (HR, 1.144; 95% CI, 1.070-1.223; *P* < .001), and colorectal cancer (HR, 1.218; 95% CI, 1.164-1.275; *P* < .001) (eFigure 2 in the Supplement). Compared with Asian patients, Hispanic patients had worse OS in all 9 cancers except for ovarian and esophageal cancers (eg, prostrate: HR, 1.206; 95% CI, 1.136-1.280; *P* < .001; ovarian: HR, 1.042; 95% CI, 0.950-1.144; *P* = .38; esophageal: HR, 1.057; 95% CI, 0.939-1.189; *P* = .36) (eFigure 2 in the Supplement).

As shown in Table 3, white, black, and Hispanic patients were more likely to have poorer CSS than Asian patients (white: adjusted HR, 1.310; 95% CI, 1.283-1.338; *P* < .001; black: adjusted HR, 1.645; 95% CI, 1.605-1.685; *P* < .001; Hispanic: adjusted HR, 1.300; 95% CI, 1.266-1.334; *P* < .001). Moreover, white, black, and Hispanic patients had poorer OS than Asian patients (white: adjusted HR, 1.333; 95% CI, 1.310-1.357; *P* < .001; black: adjusted HR, 1.754; 95% CI, 1.719-1.789; *P* < .001; Hispanic: adjusted HR, 1.279; 95% CI, 1.269-1.326; *P* < .001).

**Table 3.** Associations of Race/Ethnicity With CSS and OS

Population	CSS		OS	
	HR (95% CI)	*P* value	HR (95% CI)	*P* value
Asian	1 [Reference]	NA	1 [Reference]	NA
White	1.310 (1.283-1.338)	<.001	1.333 (1.310-1.357)	<.001
Black	1.645 (1.605-1.685)	<.001	1.754 (1.719-1.789)	<.001
Hispanic	1.300 (1.266-1.334)	<.001	1.279 (1.269-1.326)	<.001

Abbreviations: CSS, cancer-specific survival; HR, hazard ratio; NA, not applicable; OS, overall survival.

## Discussion

An important feature in our study, which distinguishes it from other studies, is that it involved the comprehensive analysis of stage at diagnosis, treatment, and survival. We found that white patients were more likely than Asian patients to develop metastasis in stomach, lung, liver and/or IHBD, and colorectal cancers. We also found that black patients were more likely to have metastatic prostate, ovarian, breast, and colorectal cancers than Asian patients. A reason may be shortages of physicians and medical centers in communities of color.^13,14,15,16^

Our findings conform with studies demonstrating that Asian patients with gastric cancer have better survival than patients from other racial/ethnic groups.^17,18^ Asian patients with gastric cancer have a higher 5-year survival rate than white patients, which might be explained by routine screening practices in Asia.^19^ Screenings, such as annual or biennial upper endoscopy, in men and women aged 40 to 50 years are a routine practice.^20,21,22^ Some immigrants from Asia may have already completed screening before relocating to the United States. However, upper endoscopy is only recommended in the United States for immigrants from these high-risk endemic regions who are older than 40 years.^23^ Therefore, screening has led to significantly lower morbidity and mortality, possibly because of earlier detection and opportunities for curative resections. Population-based screening is not routinely recommended in the United States, resulting in more diagnoses in the advanced stage and thus poorer prognosis among white patients.^11,24,25^ Our study did not include data on migration of patients in and out of specific SEER registry geographic areas. Despite this limitation, many studies have revealed that Asian patients with gastric cancer have better outcomes than patients from other racial/ethnic groups.

Despite nonmetastasis and active treatment, white patients with lung cancer were associated with worse survival than Asian patients. The high prevalence of epidermal growth factor receptor (*EGFR*) variants among Asian individuals and corresponding molecular-targeted medications may account for this. The *EGFR* variant, the most common gene variation in non–small cell lung cancer, is significantly higher among Asian patients with lung cancer than among white patients.^26,27,28^ Therefore, Asian patients with lung cancer may benefit most from molecular-targeted therapy with the advent of EGFR inhibitors, which have prolonged survival rates considerably.^29,30^

White patients were more likely to develop metastatic liver cancer, less likely to receive active treatment, and more likely to have worse outcomes than Asian patients. The results indicated differences in the prognosis of liver cancer across different racial/ethnic groups because of distinct etiologies. Chronic hepatitis B infection is the driving factor for hepatocellular carcinoma in the Asian population, whereas hepatitis C infection, alcoholic liver disease, nonalcoholic fatty liver disease, and untreated metabolic and inflammatory diseases are the main contributors in the white population.^31,32,33^ The evolving obesity and nonalcoholic fatty liver disease epidemics are dominant etiologies and risks for hepatocellular carcinoma but have no promising therapy in Western countries, whereas the hepatitis B virus vaccination has attenuated hepatitis B infection in Asian countries,^34,35^ which may explain the worse outcomes for liver cancer among the white patients in our study.

The Asian patients in our study with colorectal cancer had the best survival outcomes compared with other groups. This result has a multifaceted explanation. First, it may be associated with the diverse dietary habits among different racial/ethnic groups. Excessive intake of fat, calories, and red meat and a high body mass index are likely associated with colorectal cancer among white populations.^36,37^ The mounting prevalence of anal sexual practices among younger adults in Western countries may also be associated with the phenomenon.^38^ Lower rates of condom use during anal intercourse may bring the anus and rectum into contact more than during vaginal intercourse.^39^The proximity of the rectum to the anus and the known oncogenic association of human papillomavirus with anal cancer indicates the possible role of sexually transmitted infections in colorectal cancer.^40^ There is a physiologic association of human papillomavirus with colorectal cancer; thus, high-risk sexual behaviors among younger adults may be another explanation.^41^ Finally, inflammatory bowel disease and other causes of bowel irritation are risk factors for colorectal cancer and are prevalent in white populations.^42^ In this study, social factors may have also played a significant role in colorectal cancer mortality, similar to other malignant tumors. The socioeconomic status, health insurance coverage, and access to medical care among black US residents tends to be lower than that among members of other racial/ethnic groups.^43^ Although screenings for colorectal cancer in the United States have increased, disparities in utilization across racial/ethnic groups exist, and screenings among black patients continue to lag behind.^44,45^ Black patients have a higher frequency of *KRAS* variants in tumors, thereby promoting the aggressiveness of colorectal cancer.^46,47^ These factors may have led to the lower survival among black patients with colorectal cancer.

Our study found a higher HR of mortality for white patients with prostate cancer compared with Asian patients. The active adoption of hormone therapy in Asian patients, despite their relatively older age at diagnosis, may account for their better survival.^48^ Compared with Asian patients, white patients received more treatment for ovarian cancer and had optimal prognoses for breast cancer, whereas black patients received therapy less often and experienced worse outcomes. There a few possible reasons. First, a greater genetic predisposition was found among black women with higher risk allele frequencies at the *TERT* locus and deleterious *BRCA1/2* variants compared with patients from different racial/ethnic groups.^49^ Second, black women are more likely to develop poorly differentiated tumors and have a higher incidence of basal-like and triple-negative breast cancer, which are associated with poorer prognoses.^50^ Third, studies have found that risk among black patients receiving delayed and nonstandard treatment is substantially elevated.^51,52^ Nevertheless, white women tend to have magnetic resonance imaging targeting breast cancer and genetic testing of high sensitivity. Moreover, they also undergo more aggressive preventive procedures, such bilateral mastectomies, which greatly improve their survival outcomes.^51^

### Strengths and Limitations

This study has several strengths. First, it includes comprehensive information on stage at diagnosis, treatment, and survival, which has not often been reported. Second, the study included a set of leading cancers using a large number of patients from the SEER database. Third, rigorous statistical methodology was used to ensure the study’s accuracy.

Our study has limitations. First, individuals of mixed race/ethnicity were not included in the study. Second, the associations of chemotherapy and molecular-targeted treatment with survival were not analyzed because relevant data were not available. These factors may have been associated with patients’ survival, and sensitivity to chemotherapy for patients may vary, which warrants deeper investigation. Third, data on migration, socioeconomic status, educational background, employment status, and smoking and alcohol use are not recorded in the SEER database, which may account for unexplained differences in survival.

## Conclusions

In this study, stage at diagnosis, treatment and survival were different by race and ethnicity. These findings could help to optimize treatment and improve outcomes.

## Notes

Supplement. **eFigure 1.** Differences in Stage at Diagnosis and Treatment Between Hispanic and Asian Patients With Leading Cancers **eFigure 2.** Differences in Cancer-Specific Survival and Overall Survival Between Hispanic and Asian Patients With Leading Cancers
