# Supplementary material for: Differences in Stage of Cancer at Diagnosis, Treatment, and Survival by Race and Ethnicity Among Leading Cancer Types
Source: JAMA Netw Open. 2020 Apr 8;3(4):e202950. doi: 10.1001/jamanetworkopen.2020.2950 (PMC7142383; doi:10.1001/jamanetworkopen.2020.2950)
Supplement: Supplement. — eFigure 1. Differences in Stage at Diagnosis and Treatment Between Hispanic and Asian Patients With Leading Cancers eFigure 2. Differences in Cancer-Specific Survival and Overall Survival Between Hispanic and Asian Patients With Leading Cancers [file jamanetwopen-3-e202950-s001.pdf]

Supplementary Online Content

Zhang C, Zhang C, Wang Q, Li Z, Lin J, Wang H. Differences in stage of cancer at diagnosis, treatment, and survival by race and ethnicity among leading cancer types. *JAMA Netw Open*. 2020;3(4):e202950. doi:10.1001/jamanetworkopen.2020.2950

eFigure 1. Differences in Stage at Diagnosis and Treatment Between Hispanic and Asian Patients With Leading Cancers

eFigure 2. Differences in Cancer-Specific Survival and Overall Survival Between Hispanic and Asian Patients With Leading Cancers

This supplementary material has been provided by the authors to give readers additional information about their work.

© 2020 Zhang C et al. *JAMA Network Open.*

eFigure 1. Differences in Stage at Diagnosis and Treatment Between Hispanic and Asian Patients With Leading Cancers

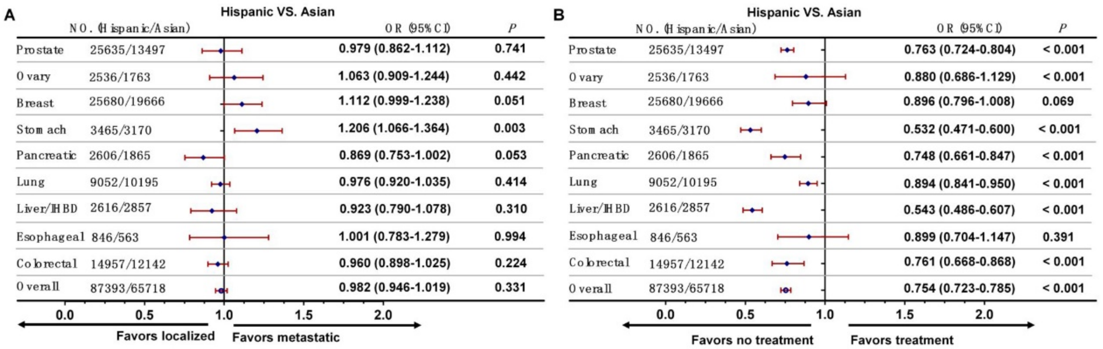

A. Forest plots depicting odds ratios (ORs) and 95% confidence intervals (CIs) for the associations between race/ethnicity (Hispanic versus Asian patients) and presentation with metastasis for each of the nine cancers evaluated. Note: Gender-specific cancers, such as prostate, breast, and ovarian cancers were not included in the overall analysis. B. Forest plots depicting odds ratios and 95% confidence intervals for the associations between race/ethnicity (Hispanic versus Asian patients) and the use of definitive therapy for each of the nine cancers evaluated. Note: Gender-specific cancers, such as prostate, breast, and ovarian cancers were not included in the overall analysis.

© 2020 Zhang C et al. *JAMA Network Open.*

eFigure 2. Differences in Cancer-Specific Survival and Overall Survival Between Hispanic and Asian Patients With Leading Cancers

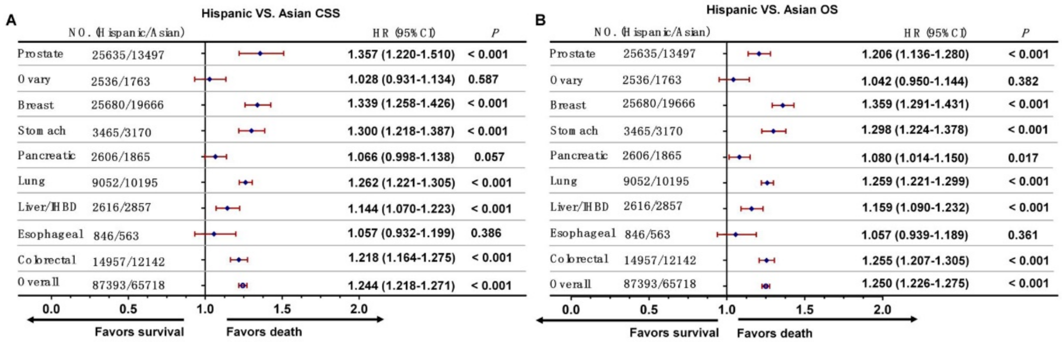

A. Forest plots depicting hazard ratios and 95% confidence intervals for the associations between race/ethnicity (Hispanic versus Asian patients) and cancer-specific survival for each of the nine cancers evaluated. Note: Gender-specific cancers, such as prostate, breast, and ovarian cancers were not included in the overall analysis. B. Forest plots depicting hazard ratios and 95% CIs for the associations between race/ethnicity (Hispanic versus Asian patients) and overall survival for each of the 9 cancers evaluated. Note: Gender-specific cancers, such as prostate, breast, and ovarian cancers were not included in the overall analysis.

© 2020 Zhang C et al. *JAMA Network Open.*
